# Supplementary material for: Analysis of the Candidate Genes and Underlying Molecular Mechanism of P198, an RNAi-Related Dwarf and Sterile Line
Source: Int J Mol Sci. 2023 Dec 22;25(1):174. doi: 10.3390/ijms25010174 (PMC10778984; doi:10.3390/ijms25010174)
Supplement: Supplementary file 1 [file ijms-25-00174-s001.zip › Figures S1-S4.pdf]

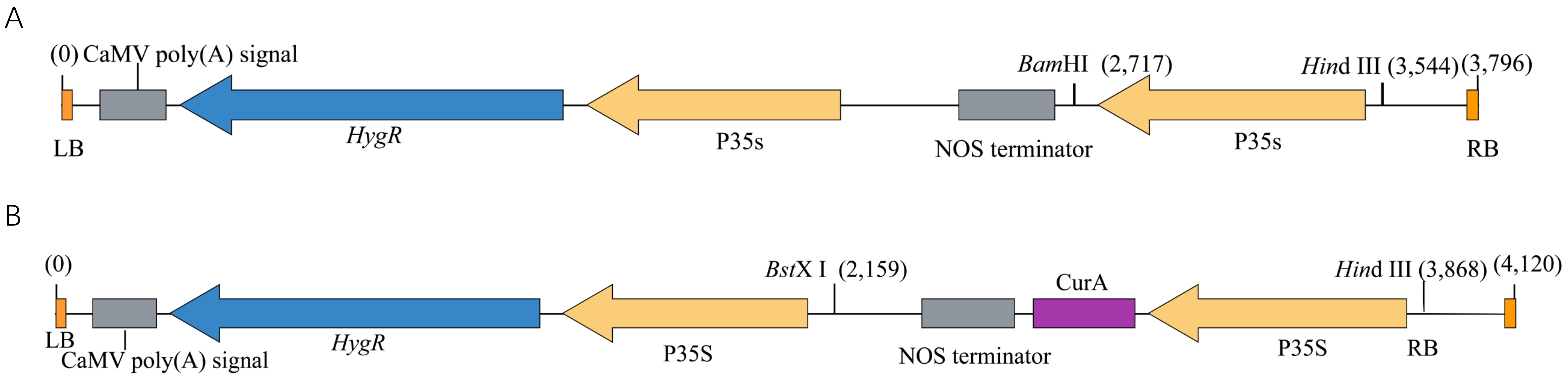

Figure S1. The schematic map of the features in T-DNA regions of vector pMDC83 (A) and pMDC83-CruA (B). The pMDC83 vector was used to construct the *B.napus* lhRNAi library, and specific *Bam*H I and *Hin*d III restriction sites were retained within the T-DNA regions. The pMDC83-CruA vector is constructed by cloning part of the *CruA* gene sequences into the vector pMDC83. The numbers in brackets indicate the position of the base pairs (bp). LB and RB, left and right T-DNA borders, respectively; HygR, *hygromycin B phosphotransferase* gene; P35S, CaMV double 35S promoter; CruA, part of *CruA* sequences containing qPCR detection sequences.

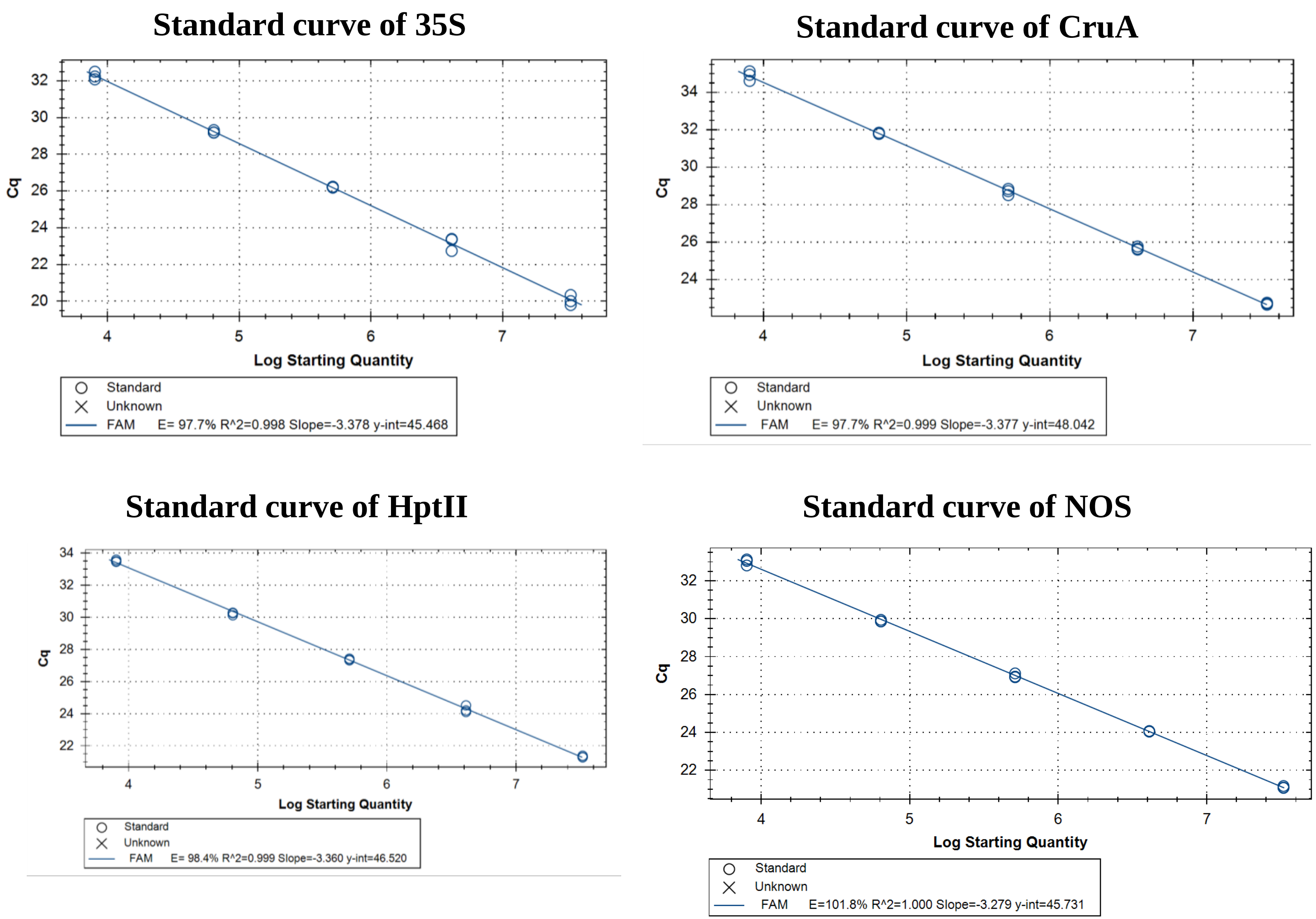

Figure S2. Standard curves for absolute quantification using qPCR. The X-axis is the logarithm of the standard plasmid concentrations, and the Y-axis is the cycle threshold for each standard plasmid. E, amplification efficiency.

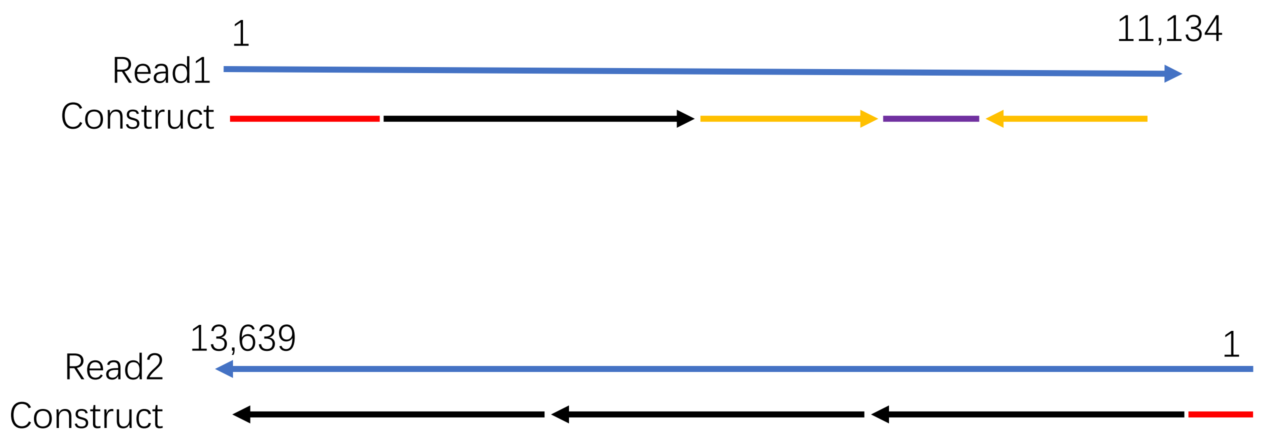

Figure S3. Schematic of the construction of two junction reads. Read1 and Read2 are junction reads mapped to 5’ and 3’ flanking sequences of inserted T-DNA in scaffoldC07, respectively. The genome and T-DNA sequences were identified with over 78% identity. Black straight arrow, T-DNA sequences from LB to RB; orange, part of T-DNA sequences from LB to RB; red line, fragment of scaffoldC07; and purple line, unknown sequences.

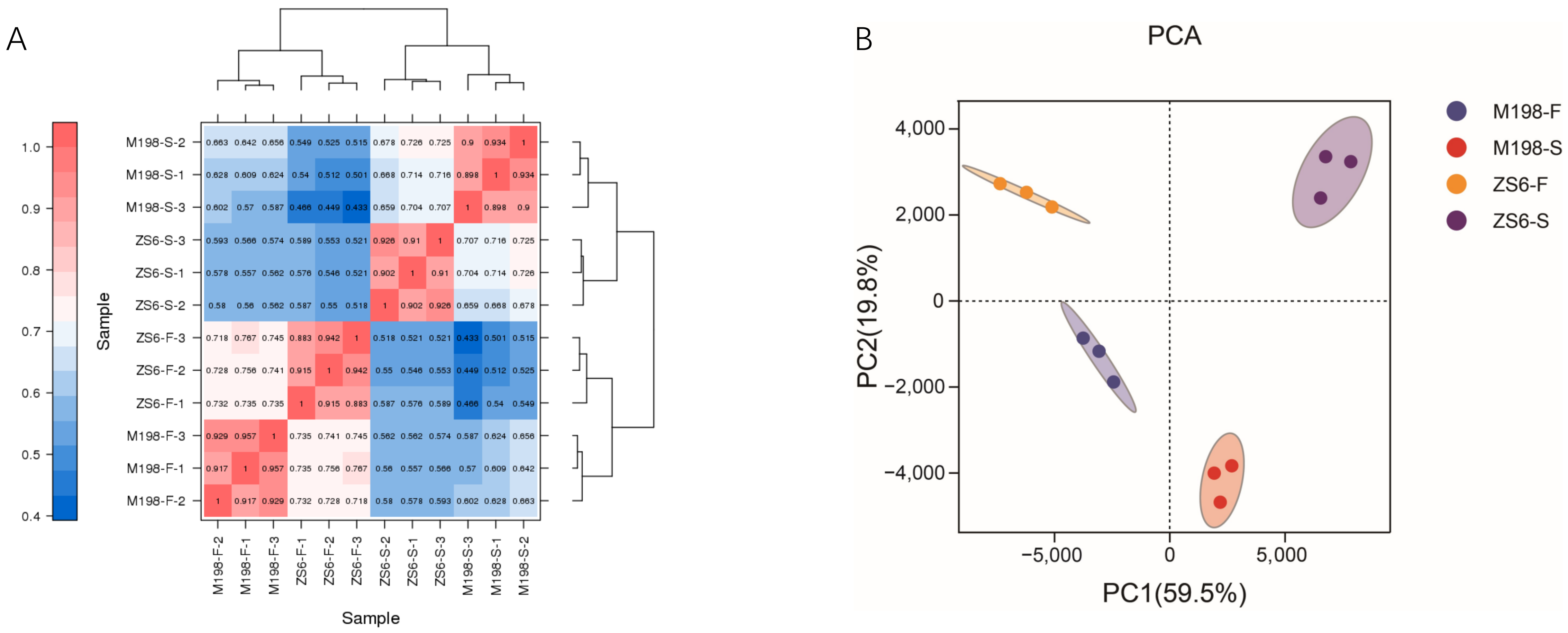

Figure S4. Correlation of normalised RNA-seq data from all biological RNA samples. (A) Pearson's correlation analysis of three replicates of various samples. (B) PCA of all biological RNA samples.
